# Exploratory Analyses of Distance, Load, and Reported Training Surface as Potential Moderators of Resisted Sled Sprint Training: A Systematic Review and Meta-Analysis

**DOI:** 10.3390/sports14090395

**Published:** 2026-09-07

**Authors:** Pablo Góngora-Rodríguez, Manuel Rodríguez-Huguet, Jorge Góngora-Rodríguez, Javier Riscart-López, Guillermo de Castro-Maqueda, Miguel Ángel Rosety-Rodríguez

**Affiliations:** 1 Department of Physical Education, School of Education Science, University of Cádiz, 11519 Puerto Real, Spain; pablo.gongora@uca.es (P.G.-R.); guillermoramon.decastro@uca.es (G.d.C.-M.); miguelangel.rosety@uca.es (M.Á.R.-R.); 2 Department of Nursing and Physiotherapy, University of Cádiz, 11009 Cádiz, Spain; jorge.gongora@uca.es; 3 Faculty of Medicine, Department of Medicine, University of Cádiz, 11003 Cádiz, Spain; javier.riscart@uca.es; 4 Salud, Deporte e Inflamación Research Group, Biomedical Research and Innovation Institute of Cádiz (INiBICA), Puerta del Mar University Hospital, University of Cádiz, Plaza Fragela, s/n, 11003 Cádiz, Spain; 5 Move-It Research Group, Biomedical Research and Innovation Institute of Cadiz, Puerta del Mar University Hospital, University of Cádiz, Plaza Fragela, s/n, 11003 Cádiz, Spain

**Keywords:** resisted sprint training, sled towing, acceleration, friction, biomechanics, athletic performance, team sports

## Abstract

This systematic review and meta-analysis investigated resisted sled training (RST) efficacy compared to unresisted sprint training (UST), alongside the moderating effects of sprint distance, sled load, and surface. Following PRISMA guidelines, PubMed, Web of Science, Scopus and SPORTDiscus were searched up to May 2026. Risk of bias was assessed via the PEDro scale. Twelve studies (N = 324) were included. Data were analyzed using Correlated Robust Variance Estimation (CRVE). Overall, RST reduced sprint times compared to UST (Hedges’ g = −0.250, *p* = 0.046; I2 = 0.0%). While time reductions were significant for early acceleration (≤10 m; g = −0.360, *p* < 0.001), and not for longer-distance outcomes (>10 m; g = −0.168, *p* = 0.293), the between-distance interaction was non-significant (*p* = 0.094). Moderate loads (g = −0.352, *p* = 0.062) and indoor gym floors (g = −0.495, *p* = 0.096) showed larger point estimates than natural grass (g = −0.180) and synthetic tracks (g = −0.197). In conclusion, no statistically significant moderating effects of distance, load, or surface were demonstrated. Because training surfaces influence the actual mechanical stimulus, prescribing sled loads based on velocity decrement (%Vdec) rather than percentage of body mass (%BM) is a promising hypothesis to standardize resistance across terrains, requiring confirmation via future prospective trials. PROSPERO: CRD420261415365.

## 1. Introduction

Linear sprinting speed and the ability to rapidly accelerate are critical determinants of athletic performance, representing the cornerstone of track and field sprinting disciplines and serving as key performance indicators in field and team sports such as soccer, rugby, and football [1,2,3]. To enhance these qualities, practitioners frequently implement unresisted sprint training (UST) [4] and resisted sprint training (RST) [5,6]. Among RST modalities, sled towing has emerged as one of the most popular methods [7]—both for track sprinters aiming to optimize their block clearance [8] and early acceleration [9], and for team-sport athletes—as it provides a mechanical overload that specifically targets the horizontal force production required during the initial phase of the sprint [10,11].

In recent years, the optimization of resisted sled training has sparked debate within the sports science literature [12]. Despite its widespread use among strength and conditioning professionals, current findings remain conflicting regarding the superiority of RST over traditional UST. While several studies report significant improvements in sprint times following sled training interventions [13], others suggest that RST provides no additional benefits compared to UST [14]. These diverging findings suggest that the efficacy of resisted sled sprinting may not be absolute, but rather dependent on underlying methodological variables.

The lack of consensus in previous research can be primarily attributed to inconsistencies in sprint distance, load allocation, and environmental conditions. Biomechanically, performance gains did not differ statistically from UST at short distances (≤10 m), which require a pronounced forward trunk lean and horizontal force application, whereas maximal velocity (>10 m) involves upright running mechanics and vertical force dominance [15]. It remains unclear if the sled overload is equally effective across both phases. Furthermore, sled loads are traditionally prescribed as a percentage of body mass (%BM) [16]. However, this approach does not account for a key physical factor: the coefficient of friction [17]. Towing 30% BM on an indoor synthetic gym floor imposes a different mechanical demand than towing the same relative load on natural grass or an athletic track, complicating cross-study comparisons [18]. While factors such as training volume, athlete level, intervention duration, and baseline sprint capacity are undoubtedly important moderators of training adaptations, surface friction directly alters the true mechanical overload (the actual dose) experienced by the athlete at any given %BM. Therefore, evaluating surface friction alongside load and distance is essential to accurately understand the dose–response relationship of RST.

Therefore, the primary aim of this systematic review and meta-analysis was to evaluate the overall efficacy of RST compared to UST, while specifically investigating the moderating effects of sprint distance, load magnitude, and surface friction. We hypothesized that the mechanical specificity of the sled would yield greater adaptations over short acceleration distances compared to longer sprints. Additionally, we hypothesized that surface friction would significantly influence the true mechanical stimulus, explaining part of the discrepancies observed in the literature.

## 2. Materials and Methods

This systematic review and meta-analysis was conducted in accordance with the PRISMA (Preferred Reporting Items for Systematic Reviews and Meta-Analyses) guidelines [19]. The original review protocol was prospectively registered in the PROSPERO database (Registration number: CRD420261415365).

### 2.1. Search Strategy and Inclusion Criteria

A comprehensive literature search was conducted in electronic databases, including PubMed, Web of Science, Scopus, and SPORTDiscus, from inception up to 1 May 2026. The search strategy was designed to capture all modalities of resisted running training in athletic populations, adapting the specific syntax and Medical Subject Headings (MeSH) where applicable for each database. The search was restricted to original, peer-reviewed journal articles published in English. Gray literature, trial registries, conference proceedings, and dissertations were excluded to ensure only fully reported, peer-reviewed interventions were analyzed. To complement database searches, reference lists of all identified eligible studies and systematic reviews were manually screened (backward citation searching), and forward citation tracking was performed via Google Scholar.

The complete Boolean search strings and limiters utilized for each database are detailed in Table 1.

Following the initial search, all identified records were exported to the Rayyan web application (Qatar Computing Research Institute, Doha, Qatar) for duplicate removal and blinded screening of titles and abstracts by the researchers [20]. Title and abstract screening, as well as subsequent full-text eligibility assessments, were performed independently by two reviewers. In instances where full-text articles were not directly accessible through institutional subscriptions, the authors systematically searched for pre-prints on academic networks (e.g., ResearchGate) and, if necessary, contacted the corresponding authors directly via email to request the manuscript. Ultimately, all required full texts were successfully retrieved. Any discrepancies or disagreements during the selection process were resolved through discussion and consensus, or through consultation with a third reviewer when necessary. Inter-reviewer agreement prior to consensus was high, with an overall percentage agreement of 93% (Cohen’s κ = 0.85) during full-text evaluation.

Eligibility criteria were defined according to the PICOS framework, as pre-registered in the PROSPERO protocol:

(P) Population: Healthy, trained, or competitive athletes (of any sex), including track and field athletes (sprinters, middle, and long-distance runners), trail runners, and competitive team-sport players with a documented training routine. Sedentary individuals, clinical populations, injured athletes, children under 15 without a formal competitive background, and animal models were strictly excluded.

(I) Intervention: Resisted running training. The original PROSPERO-registered protocol encompassed all modalities of resisted sprint training (e.g., sled towing, parachutes, tethered running, uphill sprinting). However, following the systematic screening phase—and strictly prior to data extraction and statistical analysis—the selection process yielded a sufficient number of homogeneous studies (*K* = 12) solely for sled towing. Consequently, to ensure statistical power and methodological rigor, the scope of this quantitative synthesis was refined post hoc to focus specifically on horizontal sled towing versus unresisted sprinting. Although this narrowing of scope was not entered as a formal amendment to the PROSPERO protocol, it represents the only major deviation from the initially registered review.

(C) Comparison: Unresisted sprint training (UST) protocol.

(O) Outcomes: Athletic performance metrics (sprint times and velocities).

(S) Study design: Randomized and non-randomized controlled trials conducted in both field-based settings (e.g., athletics tracks, sports fields) and laboratory-based environments. Studies conducted in clinical or rehabilitation settings were excluded.

### 2.2. Data Extraction and Quality Assessment

Data were independently extracted into a standardized spreadsheet by two reviewers. While the initial search encompassed various resistance modalities, data extraction for the present analysis isolated studies comparing horizontal sled towing versus unresisted sprinting to evaluate specific methodological moderators. The extracted information included: study authors, year of publication, sample size, and post-intervention means and standard deviations for both groups (the complete extracted dataset is available in Supplementary Table S6).

To analyze the methodological moderators of RST, data were systematically coded. Sprint distances were categorized into short-distance outcomes (≤10 m) and longer-distance outcomes (>10 m). Sled loads were systematically categorized into four specific magnitudes based on operational thresholds widely used in the mechanical profiling and resisted sprint literature [12]: Light (≤25% body mass [%BM] or ≤10% velocity decrement [%Vdec]), Moderate (>25% to <50% BM or >10% to <25% Vdec), Heavy (50% to <80% BM or 25% to 50% Vdec), and Very Heavy (≥80% BM or ≥50% Vdec). To harmonize load reporting across studies, %Vdec was prioritized as the primary criterion whenever available, as it accounts for kinematic changes regardless of surface properties. When studies only reported load as %BM, classification was assigned directly according to the corresponding %BM cut-offs. However, we explicitly acknowledge that %BM and %Vdec categories are not strictly mechanically equivalent, as the same %BM can impose varying actual mechanical loads depending on surface friction. Crucially, to account for environmental factors, studies were categorized based on the reported training surface (natural Grass, synthetic Track, or indoor Gym floors). Because primary studies did not report direct measurements of kinetic coefficients of friction, these categories served as an indirect proxy for surface conditions. Methodological quality and risk of bias of the included studies were assessed using the Physiotherapy Evidence Database (PEDro) scale [21]. While tools such as RoB 2 and ROBINS-I are the current standards for randomized and non-randomized designs respectively, the PEDro scale was selected as a pragmatic, widely recognized tool for evaluating physical training interventions in sports science. Although originally designed for randomized trials, it is routinely applied in this field to both types of study designs. To address the inherent limitations of using this scale, our quality assessment focused strictly on domain-level evaluations rather than relying on an overall aggregate or mean score. For instance, we explicitly recognized the practical impossibility of blinding athletes and coaches to the exercise modality (PEDro items 5 and 6). However, because the primary outcome (sprint time) is an objectively measured variable typically assessed via electronic timing systems, the lack of blinding is unlikely to introduce substantial measurement bias. The assessment was performed independently by two reviewers, with discrepancies resolved through consensus. A detailed domain-level assessment for every included study is provided in Supplementary Table S2.

### 2.3. Statistical Analysis

The review was conducted and reported in accordance with the Preferred Reporting Items for Systematic Reviews and Meta-Analyses (PRISMA) guidelines. Statistical analyses followed recommendations from the Cochrane Handbook for Systematic Reviews of Interventions. All analyses were performed using R software (version 4.3.0; R Foundation for Statistical Computing, Vienna, Austria) [22] within the RStudio environment (version 2023.06.1, RStudio, PBC, Boston, MA, USA) [23], primarily utilizing the metafor (version 4.4-0) and clubSandwich (version 0.5-10) packages [24,25].

Standardized mean differences (SMDs) were calculated as Hedges’ (g), which incorporates a correction for small-sample bias, to compare post-intervention sprint performance outcomes between the resisted sled training (RST) and unresisted sprint training (UST) groups. Specifically, effect sizes were calculated using post-intervention means, sample sizes, and pooled post-intervention standard deviations (SD pooled). This approach was selected because exact pre–post correlation coefficients (r) were largely unavailable across the included literature, and baseline equivalence between the RST and UST groups was confirmed. To ensure transparency, particularly given the inclusion of non-randomized designs, the baseline characteristics and statistical equivalence for all included studies are explicitly detailed in Supplementary Table S3. Furthermore, while the use of adjusted estimates, ANCOVA results, or exact change-score data is methodologically preferable, these metrics were not consistently reported by the primary studies, precluding their use in our primary meta-analysis or in a separate sensitivity analysis. Furthermore, although velocity metrics were eligible during screening, all included studies ultimately reported outcomes as sprint durations (seconds). Thus, all effect sizes naturally maintained directionality without the need for sign reversals: negative values (g < 0) consistently represent a reduction in sprint time (i.e., performance improvement). For studies that did not directly report standard deviations (SDs) but provided standard errors (SEs) or 95% confidence intervals (CIs), missing SD values were imputed using algebraic conversions recommended by the Cochrane Handbook.

All included studies utilized parallel-group designs; no crossover trials were present. Because multiple dependent effect sizes were frequently extracted from individual studies (e.g., sprint performance outcomes reported at 5 m, 10 m, and 20 m within the same participant cohort), statistical dependence among effect sizes was addressed using a random-effects meta-analytic framework with cluster-robust variance estimation (CRVE) [26].

This approach allowed the inclusion of all relevant outcomes while accounting for within-study dependence. As the true correlations among multiple sprint-distance outcomes within the same study were generally unavailable, a working intra-study correlation coefficient of (*ρ* = 0.5) was specified for the primary analysis. Cluster-robust variance estimation provides asymptotically consistent standard error estimates even when the working correlation structure is misspecified. To evaluate the robustness of the findings to this assumption, a pre-planned sensitivity analysis was conducted across a range of plausible correlation values (*ρ* = 0.2, 0.5, and 0.8).

Between-study heterogeneity was estimated using the restricted maximum likelihood (REML) estimator to obtain (*T*^2^). Given the relatively small number of independent study clusters (k = 12), the Tipton small-sample correction [27], based on the Satterthwaite approximation for degrees of freedom, was applied using the clubSandwich package. This adjustment yields more reliable confidence intervals and significance tests and improves control of Type I error rates when the number of clusters is limited.

An overall pooled effect was first estimated, after which meta-regression analyses were conducted within the CRVE framework to examine the potential moderating effects of sprint distance (≤10 m vs. >10 m), sled load magnitude, and surface friction conditions. Statistical heterogeneity was assessed using Cochran’s (Q) statistic and further quantified through estimates of (*T*^2^) and the corresponding (*I*^2^) statistic derived from the fitted random-effects model. Consistent with conventional interpretation, (*I*^2^) values of 25%, 50%, and 75% were considered indicative of low, moderate, and high heterogeneity, respectively [28].

A leave-one-out sensitivity analysis was performed by iteratively removing one study at a time to determine whether any individual study exerted a disproportionate influence on the pooled estimates. Potential publication bias was assessed through visual inspection of funnel plot asymmetry and supplemented by Egger’s regression test [29]. However, given the limited statistical power inherent to a relatively small number of studies (*k* = 12), these diagnostics were treated strictly as exploratory assessments rather than confirmatory tests. Consequently, these findings cannot rule out publication bias and were interpreted with strict caution rather than as definitive proof of its absence. Statistical significance for all analyses was established a priori at (α = 0.05) using two-tailed tests.

## 3. Results

### 3.1. Study Selection and Characteristics

**Figure 1:**
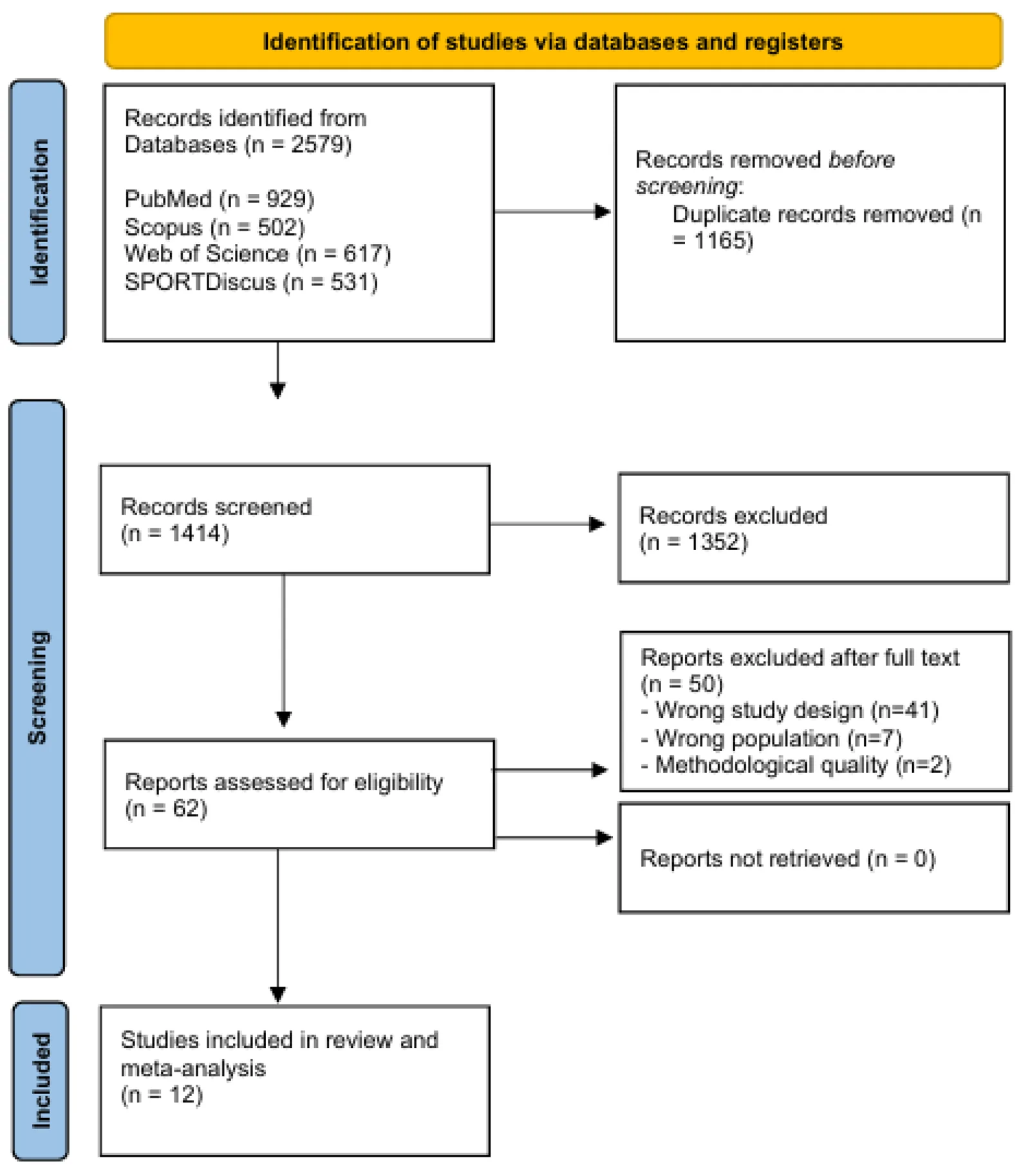
PRISMA flow diagram of the study selection process.

The initial database search yielded a total of 2579 records. After the removal of duplicates via the Rayyan web application, 1414 studies remained for title and abstract screening. Following this initial blinded screening phase, 1352 records were excluded for failing to meet basic eligibility criteria, leaving 62 full-text articles to be comprehensively assessed for eligibility. Applying the strict PICOS exclusion criteria, 50 studies were excluded for the following specific reasons: (1) ineligible intervention modality (e.g., use of non-sled resistance such as parachutes or bands; *n* = 22); (2) ineligible study design or comparator (e.g., cross-sectional designs, lack of an unresisted sprint training control group; *n* = 19); (3) ineligible population (*n* = 7); and (4) insufficient statistical reporting (e.g., missing data required for effect size calculation; *n* = 2). A complete list of full-text excluded articles along with their specific reasons for exclusion is provided in Supplementary Table S3. Ultimately, 12 studies met all inclusion criteria and were included in the systematic review and meta-analysis. The complete screening and selection process is visually detailed in the PRISMA flow diagram (Figure 1). The comprehensive methodological, participant, and intervention characteristics of all included studies—along with detailed training prescription parameters—are fully detailed in Supplementary Table S1.

### 3.2. Overall Efficacy and Moderator Analysis

**Figure 2:**
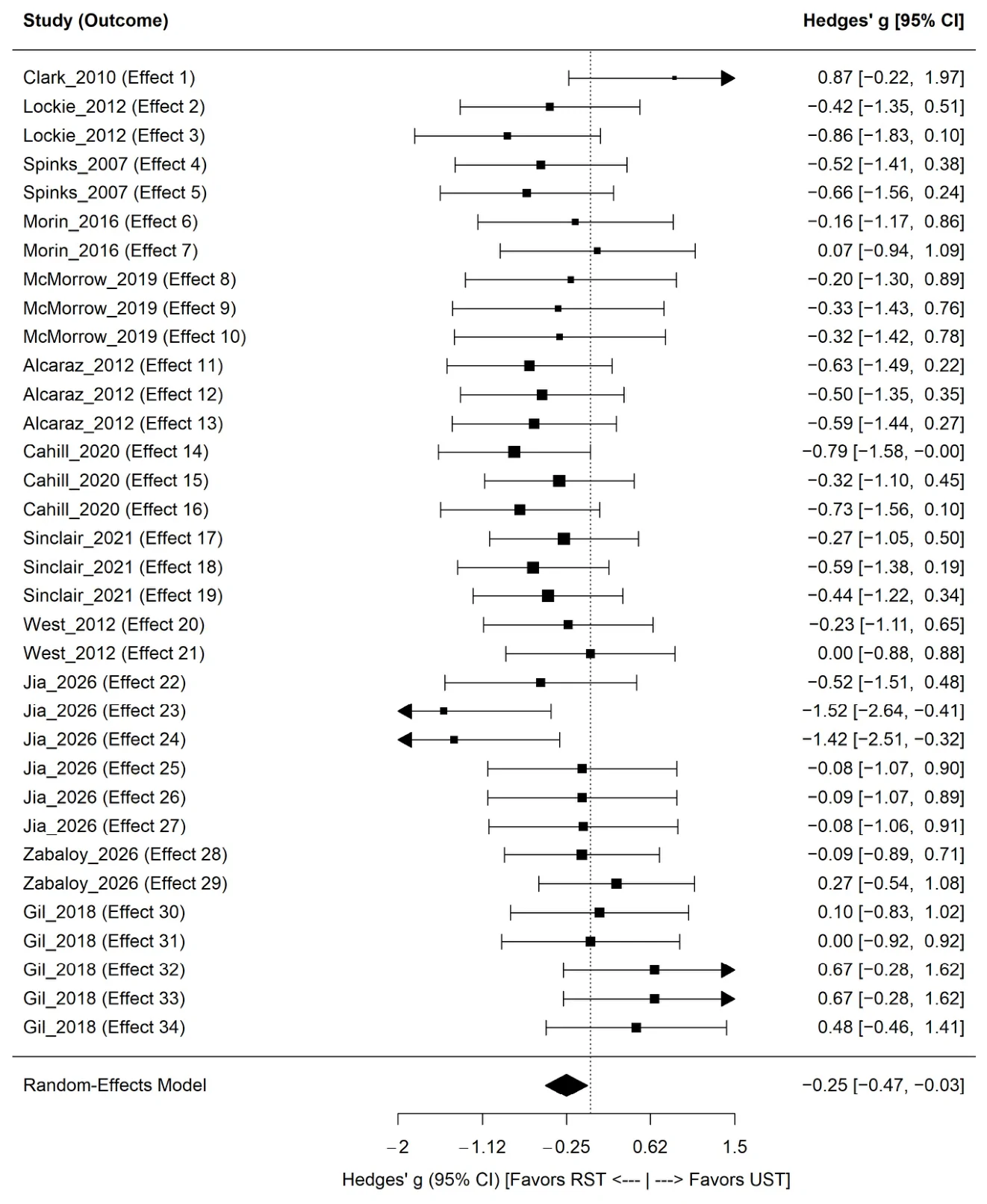
Forest plot detailing the individual study-level estimates (Hedges’ g) and the overall pooled effect of resisted sled sprint training (RST) compared to unresisted sprint training (UST). Black squares represent the individual effect sizes, and the black diamond represents the overall pooled effect. The dashed vertical line represents the overall pooled estimate, while the solid vertical line represents the null effect. Horizontal error bars denote 95% confidence intervals. Study labels (Author + Year) correspond to the primary studies included and cited within this review [13,14,30,31,32,33,34,35,36,37,38,39].

The overall model included (k = 12) independent study clusters contributing a total of (*n* = 34) effect sizes. Since the primary outcome variable extracted across all included studies was sprint time (measured in seconds), negative values for Hedges’ g denote a decrease in time, thereby representing a performance enhancement. The overall Correlated Robust Variance Estimation (CRVE) model revealed a small beneficial effect with considerable imprecision favoring RST (Hedges’ g = −0.250; 95% CI [−0.49, −0.01]; robust standard error = 0.11; Satterthwaite degrees of freedom = 10.22; *p* = 0.046). A forest plot detailing individual study-level estimates and the overall pooled estimate is presented in Figure 2.

Overall, estimated between-study heterogeneity was negligible (*T*^2^ = 0.000, *I*^2^ = 0.0%). Cochran’s Q statistic was 35.57 (*p* = 0.348). However, this zero-variance estimate based on 12 study clusters should not be interpreted as evidence of a genuine absence of heterogeneity, as statistical power to estimate variance components is inherently low with a small sample size. Furthermore, because the between-study variance (*T*^2^) was zero, the 95% prediction interval is mathematically identical to the confidence interval [−0.49, −0.01]; however, this should be interpreted with caution rather than as a definitive boundary for future true effects. Additionally, the sensitivity analysis confirmed the stability of this primary model: varying the intra-study correlation coefficient (*ρ*) yielded almost identical pooled estimates (e.g., *ρ* = 0.2: *g* = −0.261; *ρ* = 0.5: *g* = −0.250; *ρ* = 0.8: *g* = −0.233), demonstrating that the primary meta-analytic conclusions were independent of the assumed correlation structure.

#### 3.2.1. Influence of Sprint Distance

The formal omnibus test indicated that sprint distance did not statistically moderate the efficacy of RST (F(1, 7.49) = 3.67, *p* = 0.094). Descriptively, when examining the subgroups independently, RST demonstrated a significant effect on early acceleration distances (≤10 m; Hedges’ g = −0.360, 95% CI: −0.525 to −0.195, *p* < 0.001). Conversely, the estimated effect for longer sprint distances (>10 m) was smaller (Hedges’ g = −0.168, 95% CI: −0.506 to 0.170, *p* = 0.293). However, as indicated by the omnibus test, the formal interaction between these subgroups was not statistically significant. Detailed meta-regression coefficients and pairwise contrasts are presented in Table 2.

#### 3.2.2. Influence of Sled Load Magnitude

Regarding load magnitude, the omnibus test revealed no significant moderation effect across categories (F(3, 0.86) = 0.31, *p* = 0.832). Descriptively, moderate sled loads showed a trend towards larger reductions in sprint time (Hedges’ g = −0.352; 95% CI: −0.736 to 0.032; *p* = 0.062) compared to light (g = −0.188) and heavy loads (g = −0.277). Nevertheless, formal pairwise contrasts indicated that neither moderate, heavy, nor very heavy loads yielded significantly different training effects compared to the light load reference group (Table 2).

#### 3.2.3. Influence of Surface Friction

Finally, the omnibus test for reported training surfaces indicated no significant interaction between surface types and training outcomes (F(2, 2.14) = 1.32, *p* = 0.423). Sled training performed on indoor gym floors exhibited the largest descriptive effect size (Hedges’ g = −0.495), compared to natural grass (g = −0.180) or synthetic tracks (g = −0.197). However, the meta-regression confirmed that training on indoor floors or tracks did not result in statistically different adaptations compared to the natural grass reference group (Table 2).

### 3.3. Methodological Quality and Publication Bias

According to the PEDro scale assessment, the included studies presented a mean score of 4.67 out of 10, indicative of substantial methodological limitations. Domain-level judgments (Supplementary Table S2) revealed that the most commonly unmet criteria were item 5 (blinding of subjects), item 6 (blinding of therapists/coaches), and item 7 (blinding of assessors). This was expected, as blinding is practically impossible in applied sprint training interventions. Furthermore, items related to concealed allocation (item 3) were frequently absent.

To evaluate the robustness of the primary meta-analytic model to the assumed within-study correlation, a sensitivity analysis was conducted using alternative correlation values. The pooled effect size remained highly consistent across the pre-specified range: g = −0.261 at ρ = 0.2, g = −0.250 at ρ = 0.5, and g = −0.233 at ρ = 0.8. Furthermore, an independent leave-one-out sensitivity analysis confirmed that no single study cluster disproportionately influenced the overall results, with re-estimated pooled effect sizes remaining statistically significant across all iterations (Supplementary Table S4).

Similarly, the leave-one-out sensitivity analysis demonstrated that no single study cluster exerted a disproportionate influence on the summary estimates. Iterative removal of individual studies yielded pooled effect sizes ranging between g = −0.31 and g = −0.21, with all iterations maintaining robustness (Supplementary Table S5).

**Figure 3:**
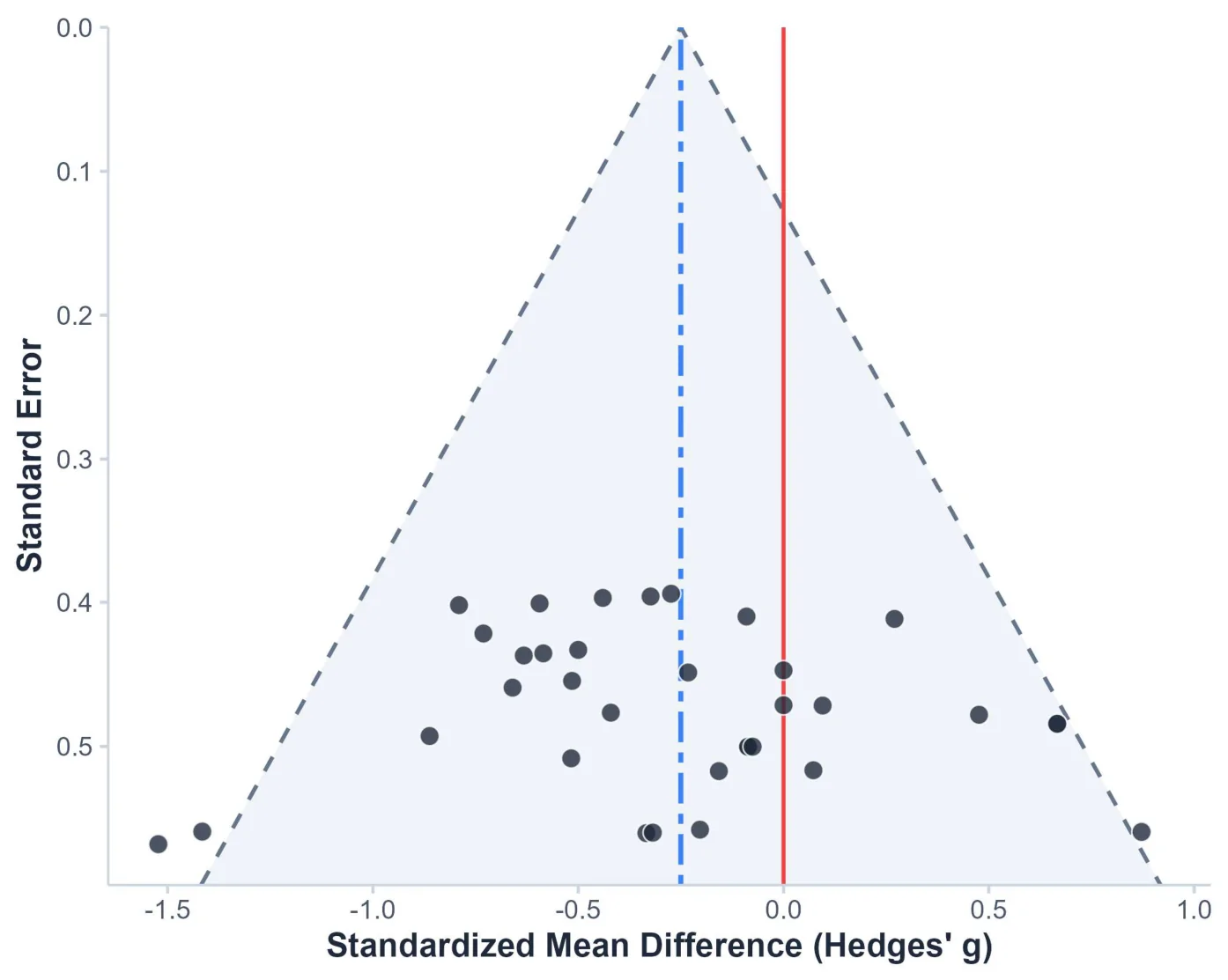
Funnel plot of standard error versus standardized mean difference (Hedges’ g) to assess publication bias. The vertical dashed blue line represents the overall pooled effect, while the solid red line indicates the null effect (0). The shaded area represents the 95% pseudo-confidence interval. Each circle represents an individual effect size from the included studies.

Visual inspection of the funnel plot (Figure 3) suggested a relatively symmetric distribution of effect sizes around the overall estimate. Egger’s regression test indicated no statistically significant funnel plot asymmetry (intercept = −1.08, SE = 4.23, 95% CI: −11.62 to 9.46, *p* = 0.808). However, this result must be interpreted with strict caution: standard Egger regression assumes independent effect sizes and exhibits reduced statistical power and potential Type I error distortion in multi-arm or dependent data structures, especially with a limited number of study clusters (k = 12), the presence of multiple dependent effects per study, and substantial methodological heterogeneity. Consequently, while no clear asymmetry was visually evident, the presence of publication bias cannot be reliably excluded.

## 4. Discussion

The primary objective of this systematic review and meta-analysis was to evaluate the overall efficacy of resisted sled training (RST) compared to unresisted sprint training (UST), and to examine potential methodological moderators of these performance adaptations. The main finding of this study is that RST provides a small beneficial effect with considerable imprecision for enhancing overall sprint times. Descriptively, our analysis indicated a statistically significant performance improvement within the early acceleration subgroup (≤10 m), whereas the effect for longer distances (>10 m) remained non-significant. However, because the formal interaction test between distance categories was not statistically significant (*p* = 0.094), these subgroup differences cannot be claimed as a confirmed moderating effect, and RST efficacy cannot be definitively categorized as phase-specific.

Before interpreting the influence of specific training variables, it must be explicitly stated that, formally, none of the three moderators (distance, load, or surface) reached statistical significance in our models. Due to the limited number of study clusters within specific categories (e.g., only two clusters for the heavy and very heavy load categories), the denominator degrees of freedom for the omnibus tests were critically low (df = 0.86 for the load omnibus test and df = 2.14 for the surface omnibus test). Under these conditions, meta-regression estimates are extremely unstable. Consequently, the ensuing discussion regarding the observed differences in point estimates between categories must be interpreted strictly as exploratory analyses. These comparisons serve to generate hypotheses for future prospective trials rather than providing definitive evidence to support immediate practical recommendations.

Although the formal meta-regression did not confirm sprint distance as a statistically significant moderator, the descriptive point estimates conceptually align with the hypothesis that mechanical specificity dictates adaptations to sled towing. In particular, a favorable training response was observed during early acceleration (≤10 m), whereas performance gains over longer distances (>10 m) were substantially attenuated and unreliable. Nevertheless, in the absence of a significant omnibus interaction, these observed differences must be framed as descriptive trends rather than conclusive evidence of a moderating influence.

These results can be interpreted through the lens of sprint biomechanics and the force–velocity profile. During early acceleration, athletes must adopt a pronounced forward trunk lean to maximize the anterior–posterior application of ground reaction forces (horizontal force) [9,40]. According to the previous literature, the resistance vector provided by a sled attached via a waist or shoulder harness directly opposes this horizontal propulsion and is theorized to create a highly specific overload that stimulates the neuromuscular system to adapt by increasing horizontal force production capability [32,41].

In contrast, across longer sprint distances (>10 m), sprinting mechanics shift dramatically: the trunk assumes an upright posture, and vertical force application becomes the primary determinant of speed [42,43]. From the perspective of previous studies, it is well documented that towing a sled—particularly over longer distances—can negatively alter running kinematics by increasing ground contact times and decreasing stride frequency [44,45,46,47,48]. Therefore, applying a horizontal resistance vector during a phzase that requires vertical force dominance lacks mechanical specificity. This may explain the attenuated and non-significant effect of RST over longer distances, where performance gains did not differ statistically from UST. In a practical context, strength and conditioning coaches seeking to specifically maximize block clearance and initial acceleration may prioritize the use of RST over short distances (typically 5 to 15 m). However, the lack of a statistically significant pooled effect on sprint times over longer distances does not preclude the use of sled towing for other valuable training objectives, such as general conditioning, technical development, or addressing athlete-specific force–velocity deficits. Therefore, RST should be systematically integrated into a comprehensive training paradigm that also adequately develops these endurance pathways.

The magnitude of the resistance represents another heavily debated variable in the literature. Our meta-analysis identified a non-significant trend where moderate sled loads (*p* = 0.062) yielded larger point estimates for sprint time reductions compared to light or heavy loads. However, lack of statistical significance precludes firm conclusions regarding the absolute superiority of moderate resistance.

Interpreting these findings requires balancing the principles of progressive overload with kinematic specificity. Light loads (typically defined as <25% BM or resulting in <10% velocity decrement [%Vdec]) are frequently prescribed to avoid disrupting sprint technique [49,50], but may fail to provide a sufficient mechanical stimulus beyond standard UST [51].

Conversely, the use of very heavy loads (e.g., ≥80% BM or ≥50% Vdec) has gained considerable traction in recent years, primarily because they maximize the mechanical demands placed on the horizontal force-generating musculature (i.e., gluteals and hamstrings) [52,53]. While biomechanical studies indicate that heavy RST is highly effective for shifting the force–velocity profile towards higher force production [54], our findings indicate that it may not always translate efficiently to overall sprint time improvements. This discrepancy is likely due to the profound acute alterations heavy sleds impose on sprint mechanics, including drastically increased ground contact times, reduced flight times, and a loss of elastic energy return [32].

Thus, moderate loads may represent a theoretical balance between force stimulus and kinematic preservation, though this premise warrants further empirical confirmation. While our pooled data reflect only overall sprint performance, external biomechanical evidence suggests that moderate loads apply sufficient horizontal overload to induce neuromuscular adaptations while allowing the athlete to maintain a high rate of force development (RFD) and proper technical execution. Heavy loads should not be discarded, but rather periodized strategically or reserved for athletes with specific horizontal force deficits (e.g., team-sport athletes needing to overcome external resistance, such as rugby forwards) [13,32].

A novel and frequently ignored aspect of RST addressed in this review relates to terrain interaction, which is evaluated here using the reported training surface as an exploratory proxy for surface friction [18,55]. Descriptively, sled training performed on indoor gym floors exhibited larger descriptive effect sizes (*g* = −0.495) than on natural grass (*g* = −0.180) or synthetic tracks (*g* = −0.197), although these subgroup differences lacked statistical significance (*p* > 0.05).

From a mechanical perspective, these results can be interpreted through the basic laws of friction, where the actual horizontal resistance (*F_f_*) opposing the athlete is a function of the coefficient of friction (*μ*) between the sled skates and the terrain, multiplied by the normal force (the weight of the sled). When practitioners prescribe load solely as a percentage of body mass (%BM), they are only controlling the normal force, completely ignoring *μ* [56,57]. For instance, towing a sled loaded to 30% BM on an indoor synthetic court requires greater horizontal force production to overcome surface resistance, whereas the exact same sled on a smooth synthetic track slides with significantly less resistance [18,58]. Consequently, what is labeled as a “moderate load” in one study could actually function as a “heavy or maximal load” in another, simply because of the terrain.

To resolve this potential source of variability, we hypothesize that prescribing load via velocity decrement (%Vdec) could provide a potentially more individualized prescription method, particularly when equipment and surface conditions vary, as %Vdec directly reflects true kinematic impairment regardless of surface friction [59,60,61]. Nevertheless, prescribing load as a percentage of body mass (%BM) remains highly accessible for practitioners and may still be practically useful, especially when calibrated locally against velocity decrement or force measurements for a specific athlete and terrain. However, because no direct comparative trials have prospectively evaluated %BM versus %Vdec prescription strategies under controlled conditions, this recommendation remains a theoretical hypothesis that requires experimental validation.

Beyond surface friction and load magnitude, several other methodological factors likely contributed to between-study heterogeneity. Differences in intervention duration, athlete training status and competitive level (recreationally trained vs. elite sprinters or team-sport players), and sex distribution represent important sources of variability. Furthermore, variations in sprint testing protocols—such as starting posture (standing vs. block vs. flying starts) and timing equipment (single-beam vs. dual-beam photocells, radar, or GPS)—can introduce subtle measurement error and alter observed effect sizes. Although the limited number of studies precluded formal meta-regressions for all these secondary moderators, they must be considered when interpreting overall outcomes.

Despite the robust insights provided by the Correlated Robust Variance Estimation (CRVE) model, this systematic review and meta-analysis is subject to several important limitations. Crucially, the small number of included studies (k = 12) severely restricted the statistical power of our moderator analyses and meta-regressions. As detailed in Table 2, several moderator categories are represented by a very small number of independent studies (e.g., heavy and very heavy loads). This low power resulted in wide confidence intervals and heightened uncertainty around subgroup estimates. Consequently, these subgroup comparisons must be considered strictly exploratory, and non-significant outcomes should be interpreted as inconclusive rather than definitive evidence of no effect. Additionally, included cohorts consisted predominantly of male athletes, limiting direct generalizability to elite female populations. Furthermore, the lack of detailed reporting regarding exact sled materials and terrain maintenance conditions prevented the direct calculation of mathematical friction coefficients. Moreover, to accommodate the available literature, our load classification combined %BM and %Vdec into a single categorical moderator. Because surface friction alters the actual mechanical resistance, equating %BM cut-offs directly with %Vdec categories is not mechanically equivalent and carries a potential risk of load misclassification for studies reporting solely %BM.

Lastly, although a formal certainty-of-evidence framework (such as GRADE) was not applied to the primary outcomes of this meta-analysis, several methodological constraints must be acknowledged. Considering the indirect evaluation of surface friction and the inability to reliably preclude publication bias, confidence in the estimates is limited because of the small number of studies, risk of bias, and imprecision. Consequently, establishing definitive conclusions regarding RST efficacy requires future validation through rigorous, large-scale randomized controlled trials.

Future research should focus on conducting direct, prospective, comparative trials that evaluate different load prescription methods (e.g., %BM vs. %Vdec) within the same cohort across varying surfaces. Additionally, well-powered longitudinal studies are required to explore how friction-induced mechanical changes translate to long-term kinetic and kinematic adaptations in elite sprinters and team-sport athletes.

## 5. Conclusions

This systematic review and meta-analysis indicate that resisted sled training (RST) yields a small overall effect on sprint performance compared to unresisted sprint training (UST). The available evidence suggests that RST may provide greater benefits for short-distance acceleration outcomes (≤10 m), whereas its specific advantage over UST diminishes across longer-distance sprint outcomes (>10 m); however, it must be explicitly acknowledged that the formal difference between these distance categories was not statistically significant. Furthermore, all moderator analyses conducted in this review are strictly exploratory. Subgroup analyses revealed that moderate-load interventions and studies conducted on indoor surfaces produced larger point estimates; however, these findings were statistically imprecise and should be interpreted cautiously as descriptive trends. Because surface type was categorized as an indirect proxy rather than friction being directly measured, relying solely on relative load percentage (%BM) may introduce unintentional resistance variability across different study environments. While velocity-decrement-based prescription (%Vdec) presents a promising alternative to improve individualization and comparability across settings, its practical superiority over %BM prescription requires direct prospective evaluation. Consequently, future adequately powered randomized controlled trials directly comparing different load prescription strategies under standardized surface conditions are required to establish firm practical guidelines.

## Figures and Tables

**Table 1:** Search strategy.

Database	Search String
Pubmed	((“resisted sprint”[tiab] OR “resisted sprints”[tiab] OR “resisted sprinting”[tiab] OR “sled towing”[tiab] OR “sled training”[tiab] OR “sled sprinting”[tiab] OR “sled sprint”[tiab] OR “resisted sled”[tiab] OR (resisted[tiab] AND running[tiab])) OR (tethered[tiab] AND (sprint[tiab] OR sprints[tiab] OR sprinting[tiab] OR running[tiab])) OR (parachute[tiab] AND (sprint[tiab] OR sprints[tiab] OR sprinting[tiab] OR running[tiab])) OR “horizontal resistance training”[tiab] OR “uphill sprint”[tiab] OR “uphill sprinting”[tiab] OR “uphill running”[tiab] OR (uphill[tiab] AND sprints[tiab]) OR (hill[tiab] AND (sprint[tiab] OR sprints[tiab] OR sprinting[tiab] OR training[tiab])) OR ((incline[tiab] OR inclined[tiab]) AND (sprint[tiab] OR sprints[tiab] OR sprinting[tiab]))) NOT (“Hill model”[tiab] OR “Hill-type”[tiab] OR “Hill equation”[tiab] OR “insulin”[tiab] OR “airway”[tiab] OR “patient*”[tiab] OR “clinical”[tiab])
Scopus	(“resisted sprint” OR “resisted sprints” OR “resisted sprinting” OR “resisted running” OR “sled towing” OR “sled training” OR “sled sprinting” OR “sled sprint” OR “resisted sled” OR “tethered sprint” OR “tethered sprints” OR “tethered sprinting” OR “tethered running” OR “parachute sprint” OR “parachute sprints” OR “parachute sprinting” OR “parachute running” OR “horizontal resistance training” OR “uphill sprint” OR “uphill sprints” OR “uphill sprinting” OR “uphill running” OR “hill sprint” OR “hill sprints” OR “hill sprinting” OR “hill training” OR “incline sprint” OR “incline sprints” OR “incline sprinting” OR “inclined sprint” OR “inclined sprints” OR “inclined sprinting”) NOT (“Hill model” OR “Hill-type” OR “Hill equation” OR insulin OR airway OR patient* OR clinical)
SPORTDiscuss	(“resisted sprint” OR “resisted sprints” OR “resisted sprinting” OR “resisted running” OR “sled towing” OR “sled training” OR “sled sprinting” OR “sled sprint” OR “resisted sled” OR “tethered sprint” OR “tethered sprints” OR “tethered sprinting” OR “tethered running” OR “parachute sprint” OR “parachute sprints” OR “parachute sprinting” OR “parachute running” OR “horizontal resistance training” OR “uphill sprint” OR “uphill sprints” OR “uphill sprinting” OR “uphill running” OR “hill sprint” OR “hill sprints” OR “hill sprinting” OR “hill training” OR “incline sprint” OR “incline sprints” OR “incline sprinting” OR “inclined sprint” OR “inclined sprints” OR “inclined sprinting”) NOT (“Hill model” OR “Hill-type” OR “Hill equation” OR insulin OR airway OR patient* OR clinical)
Web of Science	(“resisted sprint” OR “resisted sprints” OR “resisted sprinting” OR “resisted running” OR “sled towing” OR “sled training” OR “sled sprinting” OR “sled sprint” OR “resisted sled” OR “tethered sprint” OR “tethered sprints” OR “tethered sprinting” OR “tethered running” OR “parachute sprint” OR “parachute sprints” OR “parachute sprinting” OR “parachute running” OR “horizontal resistance training” OR “uphill sprint” OR “uphill sprints” OR “uphill sprinting” OR “uphill running” OR “hill sprint” OR “hill sprints” OR “hill sprinting” OR “hill training” OR “incline sprint” OR “incline sprints” OR “incline sprinting” OR “inclined sprint” OR “inclined sprints” OR “inclined sprinting”) NOT (“Hill model” OR “Hill-type” OR “Hill equation” OR insulin OR airway OR patient* OR clinical)

Note: * indicates truncation, retrieving all terms beginning with the preceding word stem.

**Table 2:** Multilevel meta-regression models (CRVE) analyzing the moderating effects of sprint distance, sled load, and reported training surface on resisted sprint training efficacy.

Moderator & Category	*k*	*n*	β (Contrast)	Robust SE	95% CI	df	*p*-Value
Sprint Distance							
Short (≤10 m)	9	13	Reference	-	-	-	-
Long (>10 m)	11	21	0.192	0.100	[−0.042, 0.426]	7.49	0.094
Omnibus Test: F(1, 7.49) = 3.67, *p* = 0.094							
Sled Load							
Light	6	15	Reference	-	-	-	-
Moderate	4	11	−0.164	0.245	[−0.736, 0.409]	7.48	0.525
Heavy	2	4	−0.089	0.384	[−1.481, 1.304]	2.45	0.835
Very Heavy	2	4	−0.011	0.261	[−0.990, 0.969]	2.34	0.971
Omnibus Test: F(3, 0.86) = 0.31, *p* = 0.832							
Reported Training Surface							
Natural Grass	7	15	Reference	-	-	-	-
Indoor Gym	2	5	−0.316	0.175	[−1.145, 0.514]	1.82	0.226
Synthetic Track	3	14	−0.017	0.336	[−0.937, 0.904]	4.13	0.963
Omnibus Test: F(2, 2.14) = 1.32, *p* = 0.423							

Note: *k* = number of independent study clusters; *n* = number of effect sizes; β = meta-regression coefficient representing the difference between the specific category and the reference category; CI = confidence interval; SE = robust standard error (CR2); df = Satterthwaite degrees of freedom.

## Data Availability

The complete dataset used to calculate all dependent effect sizes and the full R script code utilized for the CRVE analysis are provided as Supplementary Material alongside this article.

## Supplementary Materials

The following supporting information can be downloaded at https://www.mdpi.com/article/10.3390/sports14090395/s1. PRISMA 2020 checklist; Table S1: Characteristics of all included studies; Table S2: Domain-level Risk of Bias assessment using the PEDro scale; Table S3: Baseline equivalence of sprint performance outcomes across included studies; Table S4: Sensitivity analysis of the overall effect across a range of assumed intra-study correlation coefficients (*p* = 0.1 to 0.9); Table S5: Leave-one-out sensitivity analysis evaluating the influence of individual studies on the pooled effect size; Table S6: Raw data and individual metrics extracted from the included studies for the sensitivity analysis.

## Abbreviations

The following abbreviations are used in this manuscript:

BM	Body mass
CRVE	Correlated robust variance estimation
RST	Resisted sprint training
UST	Unresisted sprint training
VDec	Velocity decrement
