# Supplementary material for: Exploratory Analyses of Distance, Load, and Reported Training Surface as Potential Moderators of Resisted Sled Sprint Training: A Systematic Review and Meta-Analysis
Source: Sports (Basel). 2026 Sep 7;14(9):395. doi: 10.3390/sports14090395 (PMC13611309; doi:10.3390/sports14090395)
Supplement: Supplementary file 1 [file sports-14-00395-s001.zip › Suplementary Tables S1-S5.pdf]

Supplementary Table S1. Characteristics of all included studies.

Study	Sport	Level	Gender	Age	Distance	Surface	Weeks	Frequency	Load
Spinks et al. (2007)	Field Sports	Club	Male	21.8	0-5m	Gym	8	2	Light
Spinks et al. (2007)	Field Sports	Club	Male	21.8	0-15m	Gym	8	2	Light
Clark et al. (2010)	Lacrosse	Collegiate	Male	19.8	18.3-54.9m	Grass	7	2	Light
Alcaraz et al. (2012)	Track and Field	Trained	Mixed	21.6	0-10m	Track	4	3	Light
Alcaraz et al. (2012)	Track and Field	Trained	Mixed	21.6	0-20m	Track	4	3	Light
Alcaraz et al. (2012)	Track and Field	Trained	Mixed	21.6	0-30m	Track	4	3	Light
Lockie et al. (2012)	Field Sports	Club	Male	22.2	0-5m	Grass	6	2	Light
Lockie et al. (2012)	Field Sports	Club	Male	22.2	0-10m	Grass	6	2	Light
West et al. (2012)	Rugby Union	Professional	Male	26.0	0-10m	Grass	6	2	Light
West et al. (2012)	Rugby Union	Professional	Male	26.0	0-30m	Grass	6	2	Light
Morin et al. (2016)	Soccer	Amateur	Male	26.5	0-5m	Grass	8	2	Very Heavy
Morin et al. (2016)	Soccer	Amateur	Male	26.5	0-20m	Grass	8	2	Very Heavy

Gil et al. (2018)	Soccer	Elite	Male	22.4	0-5m	Track	6	2	Light
Gil et al. (2018)	Soccer	Elite	Male	22.4	0-10m	Track	6	2	Light
Gil et al. (2018)	Soccer	Elite	Male	22.4	0-15m	Track	6	2	Light
Gil et al. (2018)	Soccer	Elite	Male	22.4	0-20m	Track	6	2	Light
McMorrow et al. (2019)	Soccer	Professional	Male	23.3	0-5m	Grass	6	2	Moderate
McMorrow et al. (2019)	Soccer	Professional	Male	23.3	0-10m	Grass	6	2	Moderate
McMorrow et al. (2019)	Soccer	Professional	Male	23.3	0-20m	Grass	6	2	Moderate
Cahill et al. (2020)	High School	Youth	Male	16.9	0-20m	Grass	8	2	Moderate
Cahill et al. (2020)	High School	Youth	Male	16.9	0-20m	Grass	8	2	Very Heavy
Cahill et al. (2020)	High School	Youth	Male	16.9	0-20m	Grass	8	2	Very Heavy
Sinclair et al. (2021)	Rugby League	Elite	Male	18.8	0-5m	Gym	8	2	Moderate
Sinclair et al. (2021)	Rugby League	Elite	Male	18.8	0-10m	Gym	8	2	Moderate
Sinclair et al. (2021)	Rugby League	Elite	Male	18.8	0-20m	Gym	8	2	Moderate
Jia et al. (2026)	Sprinting	Regional	Male	16.0	0-30m	Track	8	2	Moderate

Jia et al. (2026)	Sprinting	Regional	Male	15.0	0-30m	Track	8	2	Moderate
Jia et al. (2026)	Sprinting	Regional	Male	16.0	0-30m	Track	8	2	Heavy
Jia et al. (2026)	Sprinting	Regional	Male	16.0	0-60m	Track	8	2	Moderate
Jia et al. (2026)	Sprinting	Regional	Male	15.0	0-60m	Track	8	2	Moderate
Jia et al. (2026)	Sprinting	Regional	Male	16.0	0-60m	Track	8	2	Heavy
Zabaloy et al. (2026)	Rugby	Highly Trained	Male	17.3	0-10m	Grass	4	3	Heavy
Zabaloy et al. (2026)	Rugby	Highly Trained	Male	17.3	0-30m	Grass	4	3	Heavy

Supplementary Table S2. *Domain-level Risk of Bias assessment using the PEDro scale.*

**Study (Author, Year)**	**1**	**2**	**3**	**4**	**5**	**6**	**7**	**8**	**9**	**10**	**11**	**Total Score (/10)**
Spinks et al. (2007)	1	0	0	1	0	0	0	1	0	1	1	4
Clark et al. (2010)	1	1	0	1	0	0	0	0	0	1	1	5
Alcaraz et al. (2012)	1	1	0	1	0	0	0	1	0	1	1	5
Lockie et al. (2012)	1	0	0	1	0	0	0	1	0	1	1	4
West et al. (2012)	1	0	0	1	0	0	0	1	0	1	1	4
Morin et al. (2016)	1	1	0	1	0	0	0	0	0	1	1	4
Gil et al. (2018)	1	1	0	1	0	0	0	1	0	1	1	5
McMorrow et al. (2019)	1	0	0	1	0	0	0	1	0	1	1	3
Cahill et al. (2020)	1	1	0	1	0	0	0	1	0	1	1	5
Sinclair et al. (2021)	1	1	0	1	0	0	0	1	0	1	1	6

Study (Author, Year)	1	2	3	4	5	6	7	8	9	10	11	Total Score (/10)
Jia et al. (2026)	1	1	0	1	0	0	0	1	0	1	1	5
Zabaloy et al. (2026)	1	1	0	1	0	0	0	1	0	1	1	5

Supplementary Table S3. Baseline equivalence of sprint performance outcomes across included studies.

Study	Primary Outcome	RST Group (Baseline Mean ± SD)	UST Group (Baseline Mean ± SD)	Baseline Difference (*p*-value)
Spinks et al. (2007)	Sprint velocity (V0–5 m)	3.62 ± 0.25 m/s	3.51 ± 0.32 m/s	Not explicitly reported
Spinks et al. (2007)	Sprint velocity (V5–10 m)	6.41 ± 0.23 m/s	6.39 ± 0.32 m/s	Not explicitly reported
Spinks et al. (2007)	Sprint velocity (V10–15 m)	7.19 ± 0.38 m/s	6.99 ± 0.33 m/s	Not explicitly reported
Spinks et al. (2007)	Sprint velocity (V0–15 m)	5.25 ± 0.26 m/s	5.13 ± 0.33 m/s	Not explicitly reported
Clark et al. (2010)	18.3-54.9m Sprint (s)	4.453 ± 0.212	4.373 ± 0.142	Not explicitly reported
Alcaraz et al. (2012)	Sprint velocity (V0–15 m)	6.49 ± 0.47 m/s	6.49 ± 0.47 m/s	Not explicitly reported
Alcaraz et al. (2012)	Sprint velocity (V15–30 m)	8.49 ± 0.67 m/s	8.49 ± 0.67 m/s	Not explicitly reported
Alcaraz et al. (2012)	Sprint velocity (V30–50 m)	8.83 ± 0.84 m/s	8.83 ± 0.84 m/s	Not explicitly reported
Alcaraz et al. (2012)	Sprint velocity (V0–50 m)	7.27 ± 0.50 m/s	7.27 ± 0.50 m/s	Not explicitly reported
Lockie et al. (2012)	Sprint velocity (V0–5 m)	3.81 ± 0.30 m/s	3.81 ± 0.30 m/s	Not explicitly reported
Lockie et al. (2012)	Sprint velocity (V5–10 m)	6.49 ± 0.30 m/s	6.49 ± 0.30 m/s	Not explicitly reported
Lockie et al. (2012)	Sprint velocity (V0–10 m)	4.79 ± 0.31 m/s	4.79 ± 0.31 m/s	Not explicitly reported
West et al. (2012)	10-m Sprint (s)	1.74 ± 0.10	1.74 ± 0.07	0.97

West et al. (2012)	30-m Sprint (s)	4.26 ± 0.28	4.26 ± 0.28	Not explicitly reported
Morin et al. (2016)	5-m Sprint (s)	1.43 ± 0.04	1.42 ± 0.05	Not explicitly reported
Morin et al. (2016)	20-m Sprint (s)	3.54 ± 0.10	3.51 ± 0.14	Not explicitly reported
Gil et al. (2018)	5-m Sprint (s)	1.06 ± 0.1	1.06 ± 0.1	Not reported (Groups matched by COD)
Gil et al. (2018)	10-m Sprint (s)	1.79 ± 0.1	1.77 ± 0.1	Not reported (Groups matched by COD)
Gil et al. (2018)	15-m Sprint (s)	2.48 ± 0.1	2.42 ± 0.1	Not reported (Groups matched by COD)
Gil et al. (2018)	20-m Sprint (s)	3.09 ± 0.1	3.02 ± 0.1	Not reported (Groups matched by COD)
Gil et al. (2018)	25-m Sprint (s)	3.70 ± 0.1	3.62 ± 0.1	Not reported (Groups matched by COD)
McMorrow et al. (2019)	5-m Sprint (s)	1.179 ± 0.049	1.186 ± 0.077	0.84
McMorrow et al. (2019)	10-m Sprint (s)	1.940 ± 0.069	1.927 ± 0.069	0.74
McMorrow et al. (2019)	20-m Sprint (s)	3.215 ± 0.087	3.188 ± 0.076	0.57
Cahill et al. (2020)	5-m Sprint (s)	Light: 1.57 ± 0.15<br>Moderate: 1.63 ± 0.13<br>Heavy: 1.62 ± 0.10	1.6 ± 0.12	$p > 0.05$ (Groups matched by 20-m sprint speed)
Cahill et al. (2020)	10-m Sprint (s)	Light: 2.36 ± 0.18<br>Moderate: 2.45 ± 0.18<br>Heavy: 2.42 ± 0.12	2.42 ± 0.16	$p > 0.05$ (Groups matched by 20-m sprint speed)
Cahill et al. (2020)	20-m Sprint (s)	Light: 3.73 ± 0.27<br>Moderate: 3.85 ± 0.26<br>Heavy: 3.77 ± 0.15	3.84 ± 0.24	$p > 0.05$ (Groups matched by 20-m sprint speed)

Sinclair et al. (2021)	5-m Sprint (s)	1.03 ± 0.07	1.02 ± 0.06	Not explicitly reported
Sinclair et al. (2021)	10-m Sprint (s)	1.77 ± 0.06	1.76 ± 0.08	Not explicitly reported
Sinclair et al. (2021)	20-m Sprint (s)	3.01 ± 0.10	3.03 ± 0.12	Not explicitly reported
Jia et al. (2026)	30-m Sprint (s)	25% BM: 4.144 ± 0.038<br>40% BM: 4.130 ± 0.062<br>50% BM: 4.136 ± 0.071	4.142 ± 0.063	> 0.05
Jia et al. (2026)	60-m Sprint (s)	25% BM: 7.286 ± 0.065<br>40% BM: 7.290 ± 0.131<br>50% BM: 7.272 ± 0.059	7.274 ± 0.076	Not explicitly reported
Zabaloy et al. (2026)	Sprint velocity (V0–10 m)	5.31 ± 0.23 m/s	5.31 ± 0.23 m/s	Not explicitly reported
Zabaloy et al. (2026)	Sprint velocity (V10–20 m)	7.66 ± 0.27 m/s	7.66 ± 0.27 m/s	Not explicitly reported
Zabaloy et al. (2026)	Sprint velocity (V0–30 m)	6.80 ± 0.26 m/s	6.80 ± 0.26 m/s	Not explicitly reported

Supplementary Table S4. Sensitivity analysis of the overall effect across a range of assumed intra-study correlation coefficients (p = 0.1 to 0.9)

**Omitted Study Cluster**	**Re-estimated Pooled Effect Size (g)**	**Statistical Significance**
Gil et al. (2018)	-0.311	$p < 0.05$
Clark et al. (2010)	-0.297	$p < 0.05$
Zabaloy et al. (2026)	-0.287	$p < 0.05$
Morin et al. (2016)	-0.263	$p < 0.05$
West et al. (2012)	-0.260	$p < 0.05$

Omitted Study Cluster	Re-estimated Pooled Effect Size (g)	Statistical Significance
McMorrow et al. (2019)	-0.246	$p < 0.05$
Jia et al. (2026)	-0.237	$p < 0.05$
Sinclair et al. (2021)	-0.223	$p < 0.05$
Spinks et al. (2007)	-0.220	$p < 0.05$
Lockie et al. (2012)	-0.221	$p < 0.05$
Alcaraz et al. (2012)	-0.215	$p < 0.05$
Cahill et al. (2020)	-0.205	$p < 0.05$

Supplementary Table S5. Leave-one-out sensitivity analysis evaluating the influence of individual studies on the pooled effect size

Assumed Correlation (ρ)	Pooled Effect Size (g)	95% Confidence Interval	p-value
0.1	-0.32	[-0.55, -0.09]	0.012
0.3	-0.32	[-0.55, -0.09]	0.012
0.5 (Primary)	-0.33	[-0.56, -0.10]	0.011
0.7	-0.33	[-0.56, -0.10]	0.011
0.9	-0.33	[-0.56, -0.10]	0.011

Table S6. Raw data and individual metrics extracted from the included studies for the sensitivity analysis. *(Note: Provided as a separate Excel file)*
